# Genetic Variants Associated with Body Mass Index Changes in Korean Adults: The Anseong and Ansan Cohorts of the Korean Genome and Epidemiology Study

**DOI:** 10.3390/cimb46080536

**Published:** 2024-08-19

**Authors:** Sang-Im Lee, Su-Kang Kim, Sang-Wook Kang

**Affiliations:** 1 Department of Dental Hygiene, College of Health Science, Dankook University, Cheonan 31116, Republic of Korea; hanjumuck@dankook.ac.kr; 2 Department of Biomedical Laboratory Science, Catholic Kwandong University, Gangneung 25601, Republic of Korea; 3 Department of Oral and Maxillofacial Pathology, School of Dentistry, Kyung Hee University, Seoul 02447, Republic of Korea

**Keywords:** polymorphism, obesity, body mass index, association study

## Abstract

Although previous studies have examined the relationship between obesity and genetics in response to the growing obesity epidemic, research on the relationship between obesity and long-term changes in body mass index (BMI) is limited. To investigate this relationship, data from 1030 cases in the Anseong and Ansan cohorts were collected from the Korean Genome and Epidemiology Study conducted by the Korea National Institute of Health between 2000 and 2014. Cases lacking participants’ BMI data throughout the study were excluded, resulting in a final sample size of 3074. An increase or decrease in BMI was analyzed using PLINK, STRING, and DAVID, with significant differences observed in the *AEN*, *ANKS1B*, *CSF1*, *EEF2K*, *FRAS1*, *GRIK4*, *PDGFC*, *THTPA*, and *TREH* genes. These genes were observed to cluster with pathways related to type 2 diabetes, cardiovascular disease, metabolic processes, and endocytosis-related genes. These results suggest that several genes are involved in BMI changes and that several pathways are associated with obesity risk. Moreover, some genetic variants appear to influence BMI changes in Korean adults.

## 1. Introduction

Body mass index (BMI), as termed by Ancel Keys (1904–2004) in 1972, is calculated by dividing an individual’s weight in kilograms by the square of their height in meters (kg/m^2^) [1,2]. According to the World Health Organization, BMI classifications are as follows: <18.5 is underweight, 18.5–25 is normal, 25–30 is overweight, and >30 indicates obesity (http://www.who.int, accessed on 1 August 2024).

The global obesity epidemic significantly impacts global health. Obesity, which is characterized by excessive fat accumulation in the body, contributes to various diseases through two primary mechanisms. First, increased fat mass can cause osteoarthritis, sleep apnea, and social stigmatization. Second, metabolic changes resulting from excessive fat in the body can lead to diabetes, gallbladder disease, hypertension, cardiovascular disease, and obesity-related cancers [3]. A study involving 1.46 million white adults indicated that being overweight or obese increases mortality risk due to these diseases [4].

The causes of obesity are diverse and complex. Environmental and cultural factors have significantly contributed to a sudden increase in obesity cases over the past two decades. Improved living standards have led to reduced physical activity and increased consumption [5]. Additionally, sleep endocrine disruptors and alcohol consumption have contributed significantly to this trend, garnering significant attention in medical research over the past decade [6,7,8,9]. Genetic factors related to obesity have also received considerable attention, as studies focusing on similarities among family members and adoptees raised in different environments have highlighted the hereditary nature of obesity [10]. A previous genome-wide search for genes susceptible to type 2 diabetes revealed that the obesity-related FTO gene (an alpha-ketoglutarate-dependent dioxygenase gene) is highly correlated with an increased BMI and overweight status [11]. Additionally, a single nucleotide polymorphism (SNP) in the brain-derived neurotrophic factor gene is linked to obesity [12].

In this study, we analyzed data from seven surveys conducted on the Anseong and Ansan cohorts as part of the Korean Genome and Epidemiology Study (KoGES) conducted by the Korea National Institute of Health between 2001 and 2014. The Anseong and Ansan cohort data included clinical and genetic information examining how lifestyle habits, diet, and environmental factors influenced the development of chronic diseases among residents of small and medium-sized cities between 2001 and 2014. Initially, we focused on identifying genes and genetic polymorphisms associated with long-term changes in BMI. Subsequently, we analyzed the relationships between BMI changes and the genotypes identified.

## 2. Materials and Methods

### 2.1. Participants

Data for this study were obtained from the Anseong and Ansan cohorts of KoGES, conducted by the Korea Health Research Institute. Ansan and Anseong represent rural and urban communities in Korea, respectively, with 10,030 adult participants being surveyed every alternate year between 2001 and 2014 (i.e., seven surveys). Cases in which participants discontinued participation in the surveys or in which BMI information was unavailable were excluded, resulting in 3074 cases with complete BMI data across all seven surveys. Further details regarding the Anseong and Ansan cohorts can be found in previous studies [13,14].

### 2.2. Statistical Analysis

Given that the data were presented as panel data, we assumed that the error terms remained constant over time. We estimated the fitted values using the fixed effect for each year along with regression analysis.

$${BMI}_{i,t}=\beta_{0}+{AGE}_{i,t}\beta_{1}+\gamma_{0}+\varepsilon_{i,t}$$

Here, ${BMI}_{i,t}$ represents the BMI of i from the perspective of t, and AGE_i,t_ represents the age of i from the perspective of t. γ_0_ symbolizes the fixed effect, and ε_i,t_ represents the error term.

After generating the fitted value of ${BMI}_{i,t}$ ($\hat{{BMI}_{i,t}}$), we calculated the difference $\left({{Diff}_{BMI}}_{i,t}\right)$. Specifically, we calculated the difference between $\left({{Diff}_{BMI}}_{i,7}\right)$ (year 7) and ${Diff\_BMI}_{i,1}$ (year 1) to obtain (year one) ${Delta\_BMI}_{i}$.

${Delta\_BMI}_{i}$ was then used to calculate the difference in the mean, using time as a control variable to strengthen the robustness of the findings. A positive ${Delta\_BMI}_{i}$ value indicates an increase in BMI from years 1 to 7, whereas a negative value indicates a decrease.

We conducted both full-sample and subsample analyses, dividing the samples by sex.

Table 1 summarizes the sample statistics: Panel A presents findings from the full sample, and Panel B presents findings from the subsample. In Panel A, the mean BMI was 24.607 kg/m^2^, which is within the normal range. The average age was 54 years, indicating that the sample primarily comprised older adults. The sex ratio was nearly equal, with an average sex ratio of 1. The value of $\hat{BMI}$ was 24.607, which is similar to the mean BMI. The difference in Diff_BMI was close to 0.

In Panel B, the sex-based criteria were statistically significant at the 1% significance level across all analyses. Women typically exhibited higher BMI and a wider age range, although the estimated $\hat{BMI}$ was slightly higher for men. The fact that women had a higher Diff_BMI value indicates a greater deviation than men.

After classifying the cases based on increases and decreases in BMI, an association study was conducted between these groups using PLINK (https://zzz.bwh.harvard.edu/plink/index.shtml, (accessed on 1 January 2024)) [14]. Quality control measures included the exclusion of SNPs with a call rate less than 95%, those with a minor allele frequency (MAF) below 0.05, and those that deviated from the Hardy–Weinberg equilibrium (*p* < 0.05). A correlation trend test was conducted to calculate the *p*-value and identify differences in genotype frequency between groups.

Among the statistically significant genes, only those with a *p*-value < 0.01 and their protein–protein interactions were validated using STRING [15]. Network clustering was confirmed using the Markov clustering (MCL) algorithm with an inflation parameter of 3.0. Genes were then classified separately based on clustering, and their functions were identified using DAVID [16].

### 2.3. Ethics Statement

This study was approved by the institutional review board of Kyung Hee University (KHSIRB-17-057).

## 3. Results

We examined 3074 individuals (1467 men and 1607 women) whose BMI data were available from seven surveys conducted over 14 years. Among them, 1528 individuals showed a decreasing BMI, whereas 1546 showed an increasing BMI. The degree of decrease was slightly greater in women than in men (Table 1 and Table 2).

Association studies were conducted on 77,472 SNPs across 14,676 genes. SNPs not in the Hardy–Weinberg equilibrium (*p* < 0.05) and those with minor allele frequencies less than 0.05 were removed. Ultimately, 176 SNPs from 150 genes were associated with a BMI increase in the decreasing and increasing BMI trend groups (*p* < 0.01). In men, 160 SNPs from 137 genes exhibited this association (*p* < 0.01), whereas in women, 181 SNPs from 149 genes showed this association (*p* < 0.01). Only the rs4691380 SNP of the *PDGFC* gene showed statistical significance (*p* < 0.01). There were few notable differences in the association studies between male and female patients. At a significance level of *p* < 0.05, SNPs in *AEN*, *ANKS1B*, *CSF1*, *EEF2K*, *FRAS1*, *GRIK4*, *PDGFC*, *THTPA*, and *TREH* genes were associated with BMI changes in both sexes. *AEN*, *ANKS1B*, *CSF1*, and *GRIK4* were generally associated with a decreased BMI, whereas *EEF2K*, *FRAS1*, *PDGFC*, *THTPA*, and *TREH* were associated with an increased BMI (Table 3).

**Figure 1:**
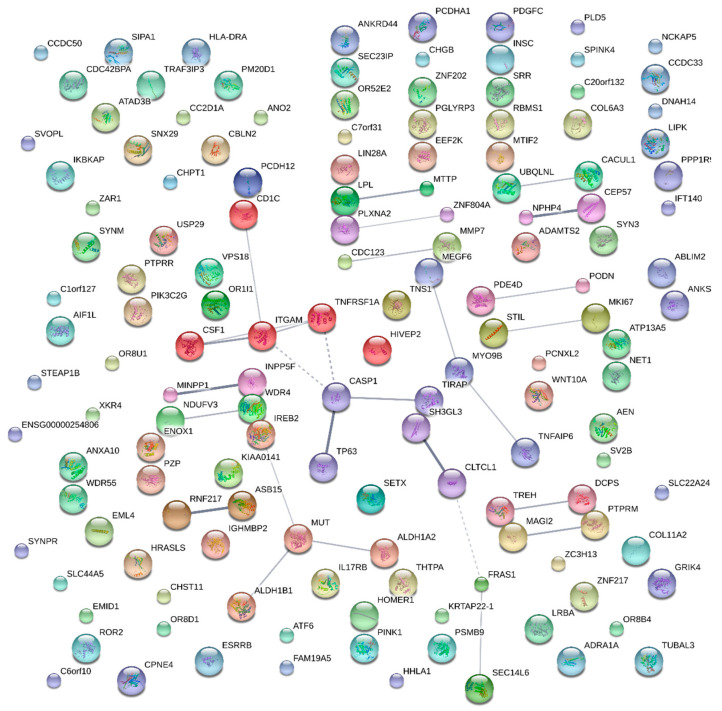
Protein–protein interactions were analyzed using the STRING program with the Markov clustering (MCL) algorithm cluster (MCL inflation parameter set to 3.0).

STRING was used to identify protein–protein interactions among 150 genes. The network comprised 143 nodes and 28 edges, with an average node degree of 0.392 and an average local clustering coefficient of 0.253. Functional enrichments within this network were not identified. As shown in Figure 1, the *CD1C*, *CSF1*, *ITGAM*, *TNFRSF1A*, *CASP1*, *TIRAP*, and *TP63* genes were clustered. DAVID was used to identify the functions of these genes, and *TIRAP*, *TNFRSF1A*, *CASP1*, *CSF1*, and *ITGAM* were associated with type 2 diabetes (Table 4). The *IREB2*, *MUT*, *ALDH1A2*, and *ALDH1B1* genes formed another cluster (Figure 1), with *MUT*, *ALDH1A2*, and *ALDH1B1* showing associations with cardiovascular disease or infection (Table 4). *SH3GL3*, *CLTCL1*, *FRAS1*, and *SEC14L6* genes were also clustered (Figure 1), and *SH3GL3* and *CLTCL1* were associated with membrane function (Table 4).

## 4. Discussion

Today, over one-third of American adults are obese, and one-third are overweight [17]. This trend is also prevalent in Korea, with a recent report indicating that 30.6% of Korean adults have a BMI over 25 [18]. An increase in obesity-related complications, such as type 2 diabetes, has also been observed [18].

Previous studies have explored the relationship between obesity and genetics [19], including many association studies on the relationship between various genes and obesity development. In this study, we analyzed changes in BMI over 14 years and identified genes affecting BMI increase or decrease. Specifically, we examined the genes of 176 SNPs with a *p*-value ≥ 0.01 using STRING and DAVID, which can visualize gene interactions. Our analyses provide important insights into the functions of specific genes or proteins and disease mechanisms.

*TIRAP*, *TNFRSF1A*, *CASP1*, *CSF1*, and *ITGAM* are associated with the development of type 2 diabetes, a condition linked to obesity [20]. *MUT*, *ALDH1A2*, and *ALDH1B1* are related to cardiovascular disease and metabolic pathways. *SH3GL3* and *CLTCL1* contribute to membrane function. Obesity increases the risk of cardiovascular disease [21]

Overall, BMI showed a decreasing trend in our study. This result contrasts with previous reports showing a decreasing trend in BMI in women aged 20–39 years but an increasing trend in men of the same age group. According to the Korean National Health and Nutrition Examination Survey, obesity prevalence among Korean men rose from 34.7% in 2005 to 39.7% in 2015, whereas it slightly decreased among women from 27.3% in 2005 to 26.0% in 2015 (http://cdc.go.kr, (accessed on 1 August 2024)). However, our results show a significant decline in obesity among men. Given that this decline is likely due to social conditions, data related to exercise habits and age should be further analyzed.

Our results highlight several notable SNPs, starting with rs4691380 of the *PDGFC* gene. PDGFC is crucial in adipogenesis and cell proliferation [22]. And it contributes to the overall regulation of fat cell formation and the expansion of adipose tissue. It is recognized as a factor associated with a BMI increase, showing significant *p* values in men (*p* = 0.003) and women (*p* = 0.000) (overall *p* = 0.000). Although research on this SNP is limited, recent studies have shown that PDGF-BB enhances adipogenesis in orbital fibroblasts [23]. rs2276064 of the *TREH* gene is a missense SNP responsible for transforming arginine into tryptophan. It is crucial in regulating trehalase activity, which is closely associated with type 2 diabetes [24] and BMI among Filipino women [25]. rs2373011 is located in the intron region of the *ANKS1B* gene, whereas rs2229165 of the *CSF1* gene has also been suggested to be involved in breast cancer etiology [26]. Studies on other SNPs are difficult to find. For rs9935059, although studies are lacking, research has shown that *eEF2K*, which is where this SNP is located, is activated during starvation (i.e., in cases of nutrient and energy depletion) [27]. Similarly, rs4982766, located in *ZFHX2*, is presumed to play a role in emotional processes, although specific studies on this SNP are unavailable [28]. Although there is a lack of previous research on rs7105363, polymorphisms in *GRIK4* have been associated with treatments for depression [29] and bipolar disorder [30]. AEN is an important mediator of p53-mediated apoptosis [31], and the *FRAS1* gene is a widely considered risk factor for Fraser syndrome [32]. Although there are no reports linking these genes to obesity, they were found to be involved with the mechanisms related to BMI changes in this study.

This study has several limitations. First, since BMI was calculated solely based on weight and height, the amount of muscle or body fat was not considered. Second, although obesity is influenced by multiple factors, only genetic factors were considered in this study. However, data from over 10,000 cases were collected, and more than 3000 of these cases, spanning seven surveys conducted over 14 years, were included and analyzed. Analyzing a large volume of data enabled us to derive more accurate results. Furthermore, in this study, we used panel analysis to predict changes in BMI over 14 years rather than at specific time points. Analyzing genes associated with changes in BMI, rather than only those related to a high BMI, can provide a more accurate interpretation of obesity. In summary, we identified several genes involved in BMI changes and the pathways associated with the risk of obesity. Some genetic variants appear to affect BMI in Korean adults. In future research, the results of this study could contribute to investigations into gene–environment interactions, the longitudinal analysis of environmental influences, and epigenetic mechanisms.

## Figures and Tables

**Table 1:** Summary of sample statistics with full sample.

Variable	Mean	Standard Deviation	Max	Min	*N*
BMI	24.607	3.032	40.259	12.535	21,518
Age	53.765	8.401	75	40	21,518
Sex	1.523	0.499	2	1	21,518
$\hat{BMI}$	24.607	0.041	24.673	24.505	21,518
Diff_BMI	0.000	3.032	15.750	−11.994	21,518

*N* refers to the total number of observation units in the panel dataset.

**Table 2:** Summary of sample statistics with subsample.

Variable	Male	Female	Difference (*t*-Value)
Age	53.329	54.164	−0.834 (−8.18) ***
BMI	24.430	24.768	−0.338 (−7.28) ***
$\hat{BMI}$	24.609	24.605	0.004 (7.28) ***
Diff_BMI	−0.179	0.163	−0.342 (−8.27) ***
*N*	10,269	11,249	Sum = 21,518

*** indicate statistical significance at 1%. *N* refers to the total number of observation units in the panel dataset.

**Table 3:** Results of an association study of single nucleotide polymorphisms associated with changes in body mass index in each group.

SNP Id	Gene	Mutations		Class	*p*-Value		
					All	Male	Female
rs7105363	*GRIK4*, *LOC105369532*	intron variant	Silent	protective	* 0.000	0.033	* 0.005
rs8027765	*AEN*	Missense	N140D	protective	* 0.000	0.010	* 0.006
rs2373011	*ANKS1B*	intron variant	Silent	protective	* 0.000	0.011	* 0.005
rs2229165	*CSF1*	intron variant, missense	Silent, G438R	protective	* 0.001	0.038	* 0.008
rs9935059	*EEF2K*	missense, transcript variant	H23R	risk	* 0.000	0.014	* 0.006
rs34670941	*FRAS1*	Missense	V3626A	risk	* 0.000	0.021	* 0.007
rs4691380	*PDGFC*	intron variant	Silent	risk	* 0.000	* 0.001	* 0.003
rs4982766	*THTPA*, *ZFHX2*	intron variant, missense	Silent, V1545A	risk	* 0.001	0.036	* 0.008
rs2276064	*TREH*	Missense	R486W	risk	* 0.001	* 0.004	0.044

* indicates statistical significance.

**Table 4:** Gene functions of clustered proteins identified using STRING.

Term	Count	%	*p*-Value	Genes
Cluster 1				
Null	4	57.1	0.006	*TNFRSF1A*, *CSF1*, *TIRAP*, *ITGAM*
Tuberculosis	3	42.9	0.009	*TNFRSF1A*, *TIRAP*, *ITGAM*
Type 2 Diabetes, edema, rosiglitazone	5	71.4	0.009	*TNFRSF1A*, *CSF1*, *TIRAP*, *CASP1*, *ITGAM*
Pharmacogenomic	5	71.4	0.020	*TNFRSF1A*, *CSF1*, *TIRAP*, *CASP2*, *ITGAM*
Unknown	4	57.1	0.029	*TNFRSF1A*, *CSF1*, *TIRAP*, *ITGAM*
Immune	4	57.1	0.139	*TNFRSF1A*, *CSF1*, *TIRAP*, *ITGAM*
Cluster 2				
Acquired immunodeficiency syndrome	3	75.0	0.014	*MUT*, *ALDH1B*, *IREB2*
Cardiovascular	4	100.0	0.036	*ALDH1A2*, *MUT*, *ALDH1B*, *IREB2*
Infection	3	75.0	0.074	*MUT*, *ALDH1B*, *IREB2*
Cluster 3				
Splice variant	3	75.0	0.150	*SH3GL3*, *FRAS1*, *CLTCL1*
Membrane	3	75.0	0.301	*SH3GL3*, *FRAS1*, *CLTCL2*
Phosphoprotein	3	75.0	0.353	*SH3GL3*, *FRAS1*, *CLTCL3*
Alternative splicing	3	75.0	0.522	*SH3GL3*, *FRAS1*, *CLTCL4*

## Data Availability

Data are contained within the article.
